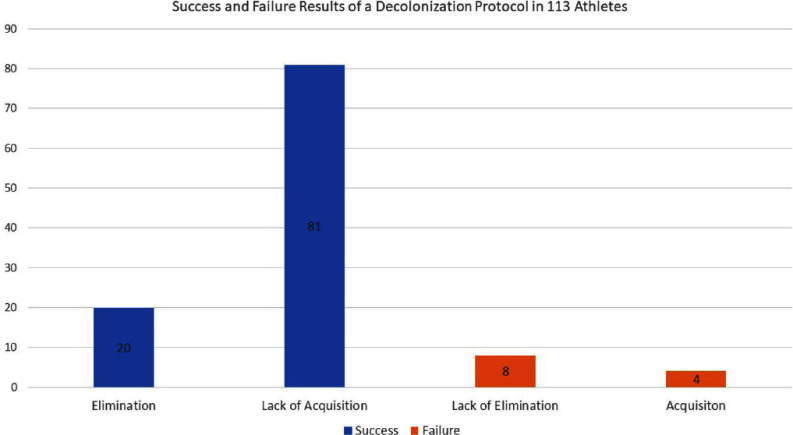# Tackling Staph: Reducing Staphylococcus aureus Colonization in a College Football Team

**DOI:** 10.1017/ash.2025.303

**Published:** 2025-09-24

**Authors:** Jessica Seidelman, Erin Gettler, Aaron Barrett, Bobby Warren, Hap Zarzour, Connor Warren, Jeffrey Bytomski, Nicholas Potter, Becky Smith, Deverick Anderson

**Affiliations:** 1 Duke University; 2 Duke University Medical Center; 3 Duke Center for Antimicrobial Stewardship and Infection Prevention; 4 Duke Center for Antimicrobial Stewardship and Infection Prevention; 5 Athletic Medicine; 6 Duke University Athletic Medicine; 7 Jeffrey Bytomski, Duke University; 8 Duke University Medical Center; 9 Duke Center for Antimicrobial Stewardship and Infection Prevention

## Abstract

**Introduction:** Athletes in contact sports have a higher rate of Staphylococcus aureus nasal carriage than the general population, leading to an increased risk of skin and soft tissue infections (SSTIs). These infections can have a significant impact on individual players and teams. This study aimed to assess the effectiveness of adding a nasal decolonization protocol in reducing S. aureus colonization among a Division I (D1) college football team to chlorhexidine gluconate body wash. **Methods:** A total of 113 athletes were screened for S. aureus nasal carriage at two time points during intensive summer training. During the first screening, athletes were universally prescribed intranasal mupirocin twice daily using clean Q-tips for five consecutive days. Players were also educated on proper hygiene and adherence to the decolonization protocol. Four weeks later, all players were screened again for S. aureus nasal carriage. Protocol success was defined as either detection of Staph aureus in the first round of screening but not in the second (elimination) or a persistently negative result (lack of acquisition). Protocol failure was defined as either the isolation of the same organism in the first and second rounds (lack of elimination) or a positive second-round culture following a negative first-round culture (acquisition). Select S. aureus isolates were submitted for multilocus sequence typing (MLST). **Results:** At the initial screening, 2 players (1.8%) were colonized with methicillin-resistant Staphylococcus aureus (MRSA), 23 players (20.4%) with methicillin-susceptible Staphylococcus aureus (MSSA), and 4 players (3.5%) with both MRSA and MSSA. After decolonization, follow-up screening identified 0 players with MRSA and 12 players (10.6%) with MSSA, representing a 58.6% reduction in overall S. aureus nasal carriage. Based on study definitions, the decolonization protocol was successful in 101 (89%) players (Figure 1).

MLST was performed on 11 of the 27 initial MSSA-positive isolates and 6 of the 12 second-round MSSA-positive isolates. Based on limited molecular typing data, at least 1 player may have acquired MSSA from another team member within the athletic environment. **Discussion:** Our findings suggest that implementing a nasal decolonization protocol in a D1 college football team is feasible and effective, resulting in a significant reduction of S. aureus nasal carriage. While initial screening effectively identified carriers, a small subset of athletes acquired MSSA colonization, indicating potential re-exposure or incomplete protocol adherence. Further research should explore decolonization adherence strategies and expand decolonization efforts across contact sports programs to reduce S. aureus-related SSTIs among athletes.

**Figure:**